# Intraoperative peripheral intravenous complications in adults: a summary of evidence for prevention and management of infiltration/extravasation

**DOI:** 10.3389/fmed.2026.1839528

**Published:** 2026-05-08

**Authors:** Hengfang Liu, Qin Zeng, Xianzhang Zeng, Xiuying Lu, Bang Xiao, Yan Cheng, Yuanfei Liu, Chun Yang, Chang Yang

**Affiliations:** 1 Department of Anesthesiology, Chongqing University Cancer Hospital, Chongqing, China; 2 Department of Operating Rooms, Sichuan Clinical Research Center for Cancer, Sichuan Cancer Hospital & Institute, Sichuan Cancer Center, Affiliated Cancer Hospital of University of Electronic Science and Technology of China, Chengdu, Sichuan, China

**Keywords:** adult, evidence-based practice, infusions, intravenous, nursing care, nursing research, patient safety, risk assessment

## Abstract

**Objective:**

To systematically synthesize the best available evidence and develop an evidence-based practice framework for the prevention, early identification, and management of intraoperative intravenous infusion complications, including non-cytotoxic drug infiltration and cytotoxic drug extravasation, in perioperative adult patients.

**Methods:**

Methodological standards for evidence summaries were strictly followed. Research questions were formulated using the PIPOST framework. Databases searched included PubMed, Embase, Web of Science, Cochrane Library, CNKI, Wanfang, SinoMed, VIP, and evidence-based platforms (UpToDate, BMJ Best Practice, JBI, GIN, NICE, RNAO). All sources were systematically searched from database inception to September 3, 2025. Following the 6S evidence pyramid, we prioritised guidelines, systematic reviews, expert consensuses, evidence summaries, and clinical decision tools over primary studies. Literature screening was conducted according to predefined inclusion and exclusion criteria. The quality of included clinical guidelines, systematic reviews, evidence summaries, expert consensus statements, and clinical decision tools was appraised using AGREE II, JBI critical appraisal tools, CASE checklists, and other appropriate instruments. Evidence was graded using the JBI evidence pre-grading system, and recommendation strength was classified as level A (strong) or level B (weak) through multidisciplinary expert consensus based on feasibility, appropriateness, clinical significance, and effectiveness.

**Results:**

Thirteen publications were included, comprising six clinical guidelines, three systematic reviews, one evidence summary, one expert consensus, and two clinical decision tools. A total of 41 evidence statements were extracted and synthesized into four thematic domains: prevention strategies; early identification and monitoring; treatment measures; and system management and improvement. Key areas included venous access planning, catheter and site selection, puncture techniques, safe administration of high-risk drugs, patient risk assessment, dynamic intraoperative monitoring principles, standardized assessment tools, and stepwise management protocols. System-level strategies such as adverse event reporting, staff training, quality improvement, and multidisciplinary collaboration were also emphasized.

**Conclusion:**

This evidence summary integrates current best evidence and provides a structured and practical framework for comprehensive perioperative management of intravenous infusion complications. Implementation of evidence-based strategies may enhance early risk detection and response, reduce complication incidence and severity, and ultimately improve patient safety and clinical outcomes.

## 1 Introduction

Intravenous infusion therapy is a fundamental and critical component of modern perioperative care, serving essential functions such as drug delivery, fluid resuscitation, blood transfusion, and nutritional support. Nevertheless, this routine procedure carries inherent risks, among which infiltration and extravasation are the most frequently encountered complications (1). Infiltration refers to the unintended leakage of non-cytotoxic fluids or medications into surrounding tissues, whereas extravasation specifically involves the escape of cytotoxic agents into perivascular spaces—this distinction is clinically significant, as extravasation can result in severe tissue injury, including necrosis, ulceration, and long-term functional impairment (2). The intraoperative environment presents unique challenges that complicate the prevention, early detection, and effective management of these events (“Anesthesia Incident Reporting System (AIRS) Case 2025–01: Leaky Pipes,” 2025) (38). Under general anesthesia or deep sedation, patients are unable to perceive or report early warning symptoms such as pain, burning, or discomfort, thereby eliminating the primary subjective indicators relied upon for timely intervention (3). Furthermore, surgical draping routinely obscures peripheral intravenous sites—commonly located on the hand, forearm, or antecubital fossa—limiting continuous visual and tactile assessment by clinicians (4). Concurrent physiological factors, including intraoperative hypotension, vasoconstriction, and limb immobilization due to positioning, may suppress classic clinical signs such as swelling or slowed infusion flow, while simultaneously increasing the risk of extravasation of vasoactive or hypertonic agents (5). Moreover, the frequent administration of high-risk substances—including anesthetics, vasopressors, hypertonic solutions, and chemotherapeutic drugs—many of which possess cytotoxic properties, significantly amplifies the potential for severe tissue damage upon extravasation compared to standard crystalloid infusions (6).

Epidemiological evidence indicates that the incidence of intraoperative peripheral catheter infiltration is clinically significant. Reported incidence rates vary: 1.43% (95% CI, 0.72–2.32%) in a meta-analysis of vasopressor extravasation (7), 2.3% (95% CI, 1.4–3.2%) in a prospective perioperative norepinephrine study (39), and 13.7% in a large systematic review of peripheral intravenous complications (8). Evidence consistently shows that extravasation risk is higher with vasoactive agents and in emergency department settings (7, 8). More concerning is the fact that, due to the concealed nature of the intraoperative setting, many infiltration and extravasation events remain undetected until the postoperative period, by which time irreversible tissue injury has often occurred (9). This delayed recognition can lead to prolonged pain, impaired wound healing, secondary infection, scarring, and in severe cases (10), necessitate surgical interventions such as debridement or flap reconstruction—outcomes that not only compromise patient recovery and satisfaction but also increase the risk of medicolegal liability (11).

Although established guidelines such as the Infusion Therapy Standards of Practice, published by the Infusion Nurses Society, offer a comprehensive framework for safe intravenous therapy (2), they do not specifically address the distinct physiological, logistical, and monitoring constraints of the operating room (12). Consequently, anesthesiologists, perioperative nurses, and surgical teams require an evidence-based, context-specific synthesis focused explicitly on intraoperative strategies for prevention, early identification, and standardized response.

The aim of this evidence summary is to systematically evaluate and integrate current literature to establish a clear, actionable practice framework for the prevention, early identification, and management of intraoperative peripheral intravenous infiltration/extravasation in adults.

To achieve this aim, we adopted a methodological approach that included systematic searching of multiple databases and evidence-based platforms, quality appraisal using validated tools (AGREE II, JBI checklists, CASE), evidence grading according to JBI criteria, and thematic synthesis of findings. The resulting framework incorporates evidence-informed strategies such as optimal catheter and site selection, utilisation of advanced technologies (e.g., near-infrared vascular imaging), structured monitoring protocols, and tiered management algorithms, with the goal of strengthening intraoperative vigilance and ultimately enhancing patient safety.

## 2 Methods

This evidence summary has been formally registered with the Evidence-Based Nursing Center of Fudan University (Registration No.: ES20258687) and is reported in full accordance with the center’s established reporting standards for evidence summaries, which align with internationally recognized frameworks including the PRISMA statement and the JBI approach for evidence synthesis.

### 2.1 Problem formulation

This study formulated the research question for the evidence summary using the PIPOST framework (13): ① P (Population): Adult patients; ② I (Intervention/Phenomenon): Prevention and management of intraoperative intravenous infusion complications, including infiltration of non-cytotoxic drugs and extravasation of cytotoxic agents; ③ P (Performer): Operating room healthcare professionals, specifically anesthesiologists and operating room nurses; ④ O (Outcome): Incidence of complications, early recognition rate, treatment success rate, and impact on patient outcomes; ⑤ S (Setting): The intraoperative and perioperative environment; ⑥ T (Type of evidence): Systematic reviews, meta-analyses, clinical practice guidelines, expert consensus statements, and published evidence summaries.

### 2.2 Evidence retrieval

This study systematically retrieved evidence by applying the computerized “6S” model of the evidence pyramid, which guided our search to prioritise higher-level evidence sources: we first searched evidence-based knowledge systems (UpToDate, BMJ Best Practice) and evidence syntheses (guidelines, systematic reviews), followed by primary studies only when higher-level evidence was insufficient. Conducting comprehensive searches across both English- and Chinese-language databases and key evidence-based resources (14). The English-language databases searched included PubMed, Embase, Web of Science, and the Cochrane Library. In addition, major evidence-based knowledge platforms and clinical guideline repositories were systematically reviewed: UpToDate, BMJ Best Practice, the JBI Evidence-Based Healthcare Centre, the Guidelines International Network (GIN), the National Institute for Health and Care Excellence (NICE), the Scottish Intercollegiate Guidelines Network (SIGN), the Registered Nurses’ Association of Ontario (RNAO), and the New Zealand Guidelines Group (NZGG). Chinese-language databases included the China National Knowledge Infrastructure (CNKI), Wanfang Data Knowledge Service Platform, the China Biomedical Literature Service System (SinoMed), and the VIP Chinese Journal Database (CQVIP). To supplement the search with up-to-date domestic guidance, Medlive was also screened for recently published clinical guidelines and expert consensus statements. The search period spanned from database inception to September 3, 2025. A combination of controlled vocabulary (e.g., MeSH, Emtree) and free-text terms was used, with search strategies adapted to align with the indexing systems and search functionalities of each individual database. Taking the PubMed database as an example, its specific search strategy is as follows:

#1 ((“Intraoperative Period”[Mesh] OR “Intraoperative Complications”[Mesh] OR (intraoperative[tiab] OR intra-operative[tiab] OR intraoperation[tiab] OR intra-operation[tiab]) OR (anesthesia[tiab] OR anaesthesia[tiab] OR anesthetic[tiab] OR anaesthetic[tiab]))AND(“Adult”[Mesh] OR adult[tiab])).

#2 (“Extravasation of Diagnostic and Therapeutic Materials”[Mesh] OR extravasat*[tiab] OR infiltrat*[tiab] OR “infusion site”[tiab] OR “IV site”[tiab])OR ((“Injections, Intravenous”[Mesh] OR “Infusions, Intravenous”[Mesh] OR IV[tiab] OR intravenous[tiab]) AND (complication*[tiab] OR injury[tiab] OR adverse[tiab])).

#3 #1 AND #2.

Following the retrieval of initial screening results, the built-in “Filters” function in PubMed was applied to restrict publication types to systematic reviews, meta-analyses, expert consensus statements, and clinical practice guidelines. The search strategies for other databases were developed using a consistent methodological approach (Please refer to the “Search Strategy.xlsx” document in the Supplementary material for details). Grey literature was not systematically searched, but reference lists of included articles and Medlive were manually screened for unpublished or in-press guidance.

### 2.3 Inclusion and exclusion criteria for evidence

#### Inclusion criteria

Population: The research subjects must be adult patients (age ≥ 18 years), with no restrictions on the type of surgery.
Intervention/phenomenon: The research topic must involve perioperative peripheral venous infusion complications, with a focus on non-cell toxic drug infiltration (such as vasoactive drugs, hypertonic fluids, contrast agents) or cell toxic drug extravasation (such as chemotherapy drugs, vesicants).
Professionals: The implementers or application targets of prevention and management measures must be members of the operating room team (such as anesthesiologists, anesthesiologist nurses, operating room nurses), and the measures must be feasible and relevant in the operating room environment.
Outcomes: The research must report on prevention strategies for complications, early identification methods, assessment means, management/treatment measures (such as physical intervention, drug antagonism, surgical intervention), or related patient safety outcomes (such as the degree of tissue damage, prognosis).
Setting: The research must take place in the intraoperative or perioperative environment (including the operating room, anesthesia preparation room, post-anesthesia care unit, and other areas directly related to the surgical process).
Type of evidence: Secondary or higher-level evidence is preferred, including systematic reviews, meta-analyses, clinical practice guidelines, expert consensus, and evidence summaries. Lower-level evidence (e.g., primary observational studies, case reports, narrative reviews) was excluded entirely, not merely deprioritised.

#### Exclusion criteria

1. Studies involving pediatric populations, including children, neonates, and infants, as well as animal studies.
2. Research primarily focused on complications associated with central venous catheters, PICC lines, or implanted ports, or studies centered on phlebitis, thrombosis, and catheter-related bloodstream infections rather than peripheral drug extravasation or infiltration.
3. Studies conducted in non-intraoperative settings, such as general wards, outpatient departments, or chemotherapy infusion centers.
4. Publications classified as narrative reviews lacking systematic methodological appraisal, case reports, small case series, commentaries, letters to the editor, conference abstracts without full-text availability, or preclinical basic science investigations.
5. Articles for which full-text access is unavailable or those not published in English or Chinese.

### 2.4 Literature screening

The literature screening was performed by two authors from our institution who had received formal training in evidence-based nursing, following a standardized and predefined protocol. The screening process consisted of two phases: initial screening based on titles and abstracts, followed by full-text assessment. Retrieved records were imported into EndNote 21, where duplicate entries were identified and removed. Two reviewers independently screened titles and abstracts to exclude studies that clearly did not meet the eligibility criteria. Full texts of potentially eligible articles were then retrieved and assessed in detail against the prespecified inclusion and exclusion criteria. Any discrepancies between reviewers were resolved through discussion, consultation of original data, or, when necessary, arbitration by a third expert in intravenous therapy nursing (15, 16).

### 2.5 Literature quality assessment

Given that the assessment tools used across the included studies varied, the specific tools adopted in this study are presented in Table 1. Prior to the formal quality assessment, all reviewers received standardized training on the application of these tools, delivered by an expert in evidence-based nursing and supplemented with illustrative examples. Only individuals who successfully completed the training were permitted to participate in the subsequent literature evaluation process. The intraclass correlation coefficient (ICC) for inter-rater reliability ranged from 0.84 to 0.92 supporting the reliability of the assessment outcomes. The detailed scoring by both raters for all included guidelines and expert consensus is provided in the Supplementary material (Scoring.xlsx).

**Table 1:** Evaluation tool table.

Type of literature	Recommended appraisal tool	Scope of application	Key domains	Appraisal criteria	Scoring method
Guidelines	AGREE II (34)	Clinical practice guidelines	Scope and purpose, stakeholder involvement, rigor of development, clarity of presentation, applicability, editorial independence	Domain score ≥60% = high quality; 30–60% = moderate quality; <30% = low quality	Each item scored on a 7-point Likert scale (1 = strongly disagree, 7 = strongly agree). Domain score = (obtained score − minimum possible score) ÷ (maximum possible score − minimum possible score) × 100%
Systematic review	JBI Critical Appraisal Checklist for Systematic Reviews and Research Syntheses (35)	Systematic reviews and meta-analyses	Methodological quality, appropriateness of review question, search strategy, criteria for inclusion, critical appraisal, data extraction, synthesis of findings.	The tool does not generate a quantitative score or quality threshold. The result is an appraisal of methodological quality and risk of bias based on the logical coherence of the “yes” responses.	Each of the 11 items is answered as Yes, No, Unclear, or Not Applicable. The appraisal is narrative and descriptive, focusing on the methodological strengths and limitations identified across the checklist items.
Summary of evidence/clinical decision	CASE (Checklist for the Appraisal of Summaries of Evidence) (36)	Evidence summaries, best practice documents, evidence-based quick reference guides	Summary development, content synthesis, presentation of recommendations, applicability.	There is no official defined quality threshold. Generally speaking, the higher the score, the better the quality. After discussion, it was agreed that literature with a score of 75% or above can be classified as high-quality literature.	Each of the 10 items is rated as Yes (1), Partial (0.5), or No (0). Total score is converted into a percentage for an overall quality grading.
Expert consensus	JBI critical appraisal checklist for textual evidence: expert opinion (35)	Expert consensus documents	Expertise, impartiality, philosophical perspective, argument consistency, incorporation of existing evidence.	The tool does not generate a quantitative score or quality threshold. The result is an appraisal of the trustworthiness and applicability of the evidence based on the logical coherence of the “yes” responses.	Each of the six items is answered as yes, no, unclear, or not applicable. The appraisal is narrative and descriptive, focusing on the strengths and limitations identified across the checklist items.

### 2.6 Evidence extraction and summary

This study included both Chinese and English literature. Initial translation was performed using Youdao Dictionary, followed by linguistic and contextual verification by nursing postgraduate students who had passed the national postgraduate English examination to ensure accuracy and reliability. Evidence extraction was conducted using a standardized structured form, with fields including title, author, publication year, study type, evidence content, and evidence level. All researchers independently completed the extraction process. Discrepancies were first resolved through re-examination of the original texts; if consensus could not be reached, a third expert in intravenous therapy was consulted for arbitration.

Evidence statements were generated using a thematic aggregation method. For evidence synthesis, the following principles were applied: First, overlapping evidence was synthesized into concise and actionable recommendations. Second, complementary findings were integrated into coherent conclusions. Third, in cases of conflicting evidence, priority was given to studies with higher levels of evidence and more recent publication dates.

The JBI evidence hierarchy (17) was used to classify evidence into five levels—Level 1 (randomized controlled trials), Level 2 (quasi-experimental studies), Level 3 (observational studies), Level 4 (descriptive studies), and Level 5 (expert opinion). For clinical guidelines and expert consensus documents, the original cited references were traced, and their respective evidence levels were retained. Recommendation strength was determined by an expert panel based on assessments of feasibility, appropriateness, clinical significance, and effectiveness, and categorized as Grade A (strong recommendation) or Grade B (weak recommendation). A Grade A recommendation required all of the following: clear evidence demonstrating that benefits significantly outweigh harms (or vice versa), high-quality supporting evidence, favorable or neutral impact on resource allocation, and full consideration of patient values and preferences. A Grade B recommendation applied when at least one of the following conditions was met: direction of effect is clear but evidence quality is low or very low, moderate confidence in benefits and harms, minimal or no negative impact on resources, or insufficient incorporation of patient values and experiences.

The expert panel consisted of seven members: two experts in evidence-based medicine and five clinical specialists—including one chief physician in surgery, three deputy chief nurses in the operating room, and one specialist in intravenous therapy—all with over 10 years of professional experience, ensuring methodological rigor and clinical relevance of the final recommendations.

## 3 Results

### 3.1 Search results

**Figure 1:**
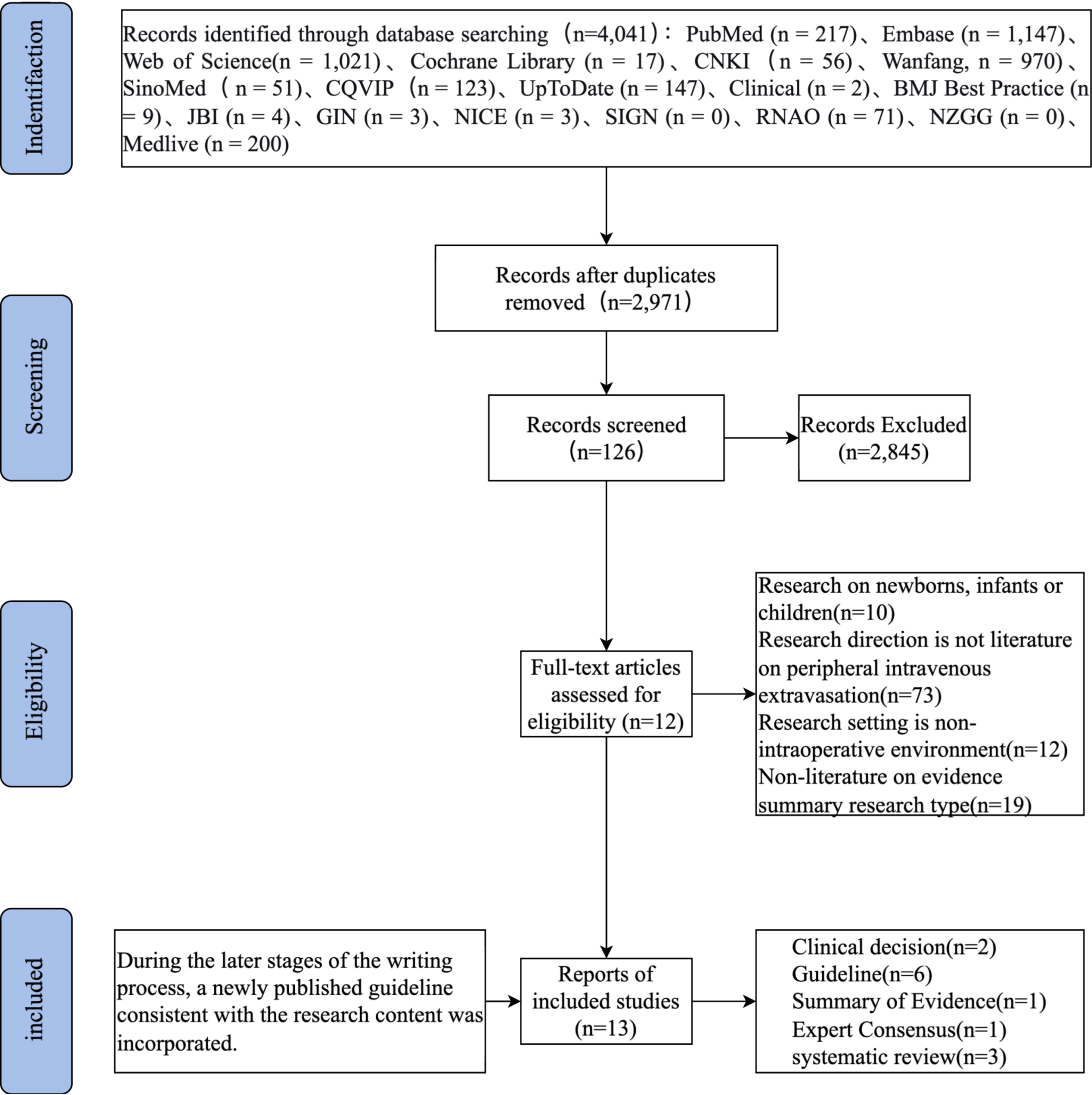
PRISMA flowchart.

This study followed the PRISMA guidelines for systematic literature search and screening. The initial search yielded 4,041 relevant articles, sourced from PubMed (*n* = 217), Embase (*n* = 1,147), Web of Science (*n* = 1,021), Cochrane Library (*n* = 17), CNKI (*n* = 56), Wanfang Data (*n* = 970), SinoMed (*n* = 51), VIP Journal (*n* = 123), UpToDate (*n* = 147), Clinical (*n* = 2), BMJ Best Practice (*n* = 9), JBI (*n* = 4), GIN (*n* = 3), NICE (*n* = 3), SIGN (*n* = 0), RNAO (*n* = 71), NZGG (*n* = 0), and Medlive (*n* = 200). After removing duplicate articles, 2,971 remained. Through initial screening based on titles and abstracts, 2,845 articles that did not meet the inclusion criteria were excluded, leaving 126 articles for full-text assessment. After a full-text review, 13 studies met all inclusion criteria and were included in this evidence summary (Table 2). During the later writing process, one additional guideline that met the criteria was identified, increasing the number of guideline-type articles to 6 (Refer to Figure 1; Table 2).

**Table 2:** General characteristics of the included literatures (*n* = 13).

Included literature	Year	Literature reference	The literature theme	Type of literature
Paulsen and Ruskin (21)	2025	UpToDate	Intravenous infusion devices for perioperative use	Clinical decision
Frank et al. (20)	2025	UpToDate	Peripheral venous access in adults	Clinical decision
Matsumoto et al. (26)	2024	JSCN, JSCO, JASP	Extravasation associated with cancer drug therapy	Guideline
Chinese Nursing Association Intravenous Therapy Professional Committee (25)	2022	CNA	Clinical Nursing Practice Guidelines for Common Complications of Intravenous Catheters	Guideline
Nickel et al. (2)	2024	INS	Infusion therapy standards of practice	Guideline
Roditi et al. (33)	2022	PubMed	Intravenous contrast medium extravasation	Guideline
Mao et al. (24)	2021	CNA	Clinical practice guideline for nursing management of iodinated contrast media extravasation	Guideline
Thompson et al. (27)	2025	ONS/ASCO	Management of Antineoplastic Extravasation	Guideline
Chen et al. (19)	2025	PubMed	Summary of best evidence for safe management of vasopressors through peripheral intravenous catheters	Summary of evidence
Thomas et al. (6)	2024	INS, AVA	Standards of C for Peripheral Intravenous Catheters: Evidence-Based Expert Consensus	Expert consensus
Tian et al. (23)	2020	PubMed	Safety of peripheral administration of vasopressor medications: A systematic review	Systematic review
Marsh et al. (8)	2020	PubMed	Peripheral intravenous catheter non-infectious complications in adults: A systematic review and meta-analysis	Systematic review
Fan et al. (22)	2023	PubMed	Relationship between indwelling site and peripheral venous catheter-related complications in adult hospitalized patients: a systematic review and meta-analysis	Systematic review

Although the systematic search identified 4,041 records, only 13 studies met the final inclusion criteria. This high attrition rate is primarily attributable to our stringent inclusion criteria, which restricted evidence to systematic reviews, meta-analyses, clinical practice guidelines, and expert consensus statements, and required explicit focus on intraoperative prevention and management of peripheral intravenous infiltration/extravasation in adults. Consequently, a large number of primary observational studies, case reports, and studies with mixed or non-intraoperative settings were excluded. While this approach ensured the inclusion of high-level evidence, it may have narrowed the evidence base and potentially excluded relevant findings published in lower-level study designs. Furthermore, publication bias – the tendency for studies with positive or definitive results to be preferentially published – cannot be ruled out, as negative or inconclusive findings on intraoperative extravasation prevention might be less likely to appear in peer-reviewed literature or guidelines. These factors should be considered when interpreting the findings of this evidence summary.

### 3.2 Quality assessment results of included literature

In the quality assessment process of this study, inter-rater consistency among all researchers exceeded 0.75 (18), indicating high agreement among evaluators and supporting the reliability of the assessment outcomes for the included literature.

#### 3.2.1 Quality assessment results of clinical decision-making and evidence summaries

Table 3 presents the quality assessment results of three studies (19–21). The two clinical decision-making articles exhibited notable limitations in search strategy and evidence grading (20, 21), whereas the evidence summary demonstrated greater methodological rigor and transparency in these domains. However, it showed some shortcomings in review process transparency (19). Overall, the evidence summary achieved the highest methodological quality and can serve as core evidence. Although the two clinical decision-making articles have limited methodological robustness, their strong clinical applicability supports their use as supplementary references, provided that their methodological limitations are explicitly acknowledged when cited.

**Table 3:** Quality assessment results of clinical decision-making and evidence summary (*n* = 3).

Evaluation items	Paulsen et al. (21)	Frank et al. (20)	Chen et al. (19)
Is the summary specific in scope and application?	Yes	Yes	Yes
Is the authorship of the summary transparent?	Yes	Yes	Yes
Are the reviewer(s)/editor(s) of the summary transparent?	Yes	Yes	No
Are the search methods transparent and comprehensive?	No	No	Yes
Is the evidence graded and is the system transparent and translatable?	No	No	Yes
Are the recommendations clear?	Yes	Yes	Yes
Are the recommendations appropriately cited?	Yes	Yes	Yes
Are the recommendations current?	Yes	Yes	Yes
Is the summary free of possible bias?	Yes	Yes	Yes
Can this summary be applied to your patient(s)?	Yes	Yes	Yes

#### 3.2.2 Quality assessment results of systematic reviews

As shown in Table 4, the three systematic reviews (8, 22, 23) demonstrated strong methodological rigor. Across 11 quality assessment items, all three studies fully met criteria in the first eight core domains—including clearly defined research questions, appropriate inclusion criteria, comprehensive search strategies, and standardized data extraction and synthesis methods—indicating a solid methodological foundation and well-documented procedures. However, for the critical ninth item, “Assessment of Publication Bias,” only the study by Fan et al. (22) explicitly conducted and reported an analysis; the studies by Tian et al. (23) and Marsh et al. (8) lacked sufficient detail or clarity, representing a key methodological gap that may compromise the robustness of their findings. Nonetheless, all three reviews derived reasonable and clinically meaningful recommendations based on available data. In sum, these systematic reviews are of high overall quality and qualify as high-quality evidence for inclusion in synthesis; however, when referencing the works of Tian DH and Marsh N, their insufficient handling of publication bias should be explicitly noted.

**Table 4:** The quality assessment results of the systematic review (*n* = 3).

Evaluation items	Tian et al. (23)	Marsh et al. (8)	Fan et al. (22)
1. Is the review question clearly and explicitly stated?	Yes	Yes	Yes
2. Were the inclusion criteria appropriate for the review question?	Yes	Yes	Yes
3. Was the search strategy appropriate?	Yes	Yes	Yes
4. Were the sources and resources used to search for studies adequate?	Yes	Yes	Yes
5. Were the criteria for appraising studies appropriate?	Yes	Yes	Yes
6. Was critical appraisal conducted by two or more reviewers independently?	Yes	Yes	Yes
7. Were there methods to minimize errors in data extraction?	Yes	Yes	Yes
8. Were the methods used to combine studies appropriate?	Yes	Yes	Yes
9. Was the likelihood of publication bias assessed?	Unclear	Unclear	Yes
10. Were recommendations for policy and/or practice supported by the reported data?	Yes	Yes	Yes
11. Were the specific directives for new research appropriate?	Yes	Yes	Yes

#### 3.2.3 Quality assessment results of guidelines

The included guidelines (2, 6, 24–26) generally demonstrated sound methodological quality. All guidelines satisfied the predefined eligibility criteria across most evaluation domains (with ≥60% and ≥30% coincidence rates, respectively). The intraclass correlation coefficient (ICC) for inter-rater reliability ranged from 0.84 to 0.92, with an overall ICC (3,1) of 0.887—indicating good agreement between raters according to established interpretation criteria and supporting the reliability of the assessment outcomes. Specifically, the guidelines by Nickel et al. (2) and Thomas et al. (6) achieved outstanding scores across all AGREE II domains, particularly in “rigor of development” and “applicability.” The guideline by Matsumoto et al. (26) showed balanced performance throughout. The guideline issued by the Intravenous Therapy Committee of the Chinese Nursing Association (25) scored highly in “scope and purpose” and “clarity of presentation,” but its “applicability” score was notably lower (33.3%), suggesting relevant limitations should be considered during implementation. Similarly, the guideline by Mao et al. (24) was comparatively weak in “applicability” (29.17%). Overall, all four guidelines met the criteria for inclusion, with those by Nickel et al. (2) and Thomas et al. (6) exhibiting the highest methodological credibility (Refer to Table 5).

**Table 5:** Quality assessment results of clinical guidelines (*n* = 6).

Guideline	Standardized scores in various fields								
	Scope and purpose	Stakeholder involvement	Rigour of development	Clarity of presentation	Applicability	Editorial independence	≥60%	≥30%	Quality evaluation
Matsumoto et al. (26)	94.4%	66.7%	79.2%	88.9%	58.3%	75%	6	6	A
Chinese Nursing Association Intravenous Therapy Professional Committee (25)	100%	72.2%	68.8%	94.4%	33.3%	83.3%	5	6	B
Mao et al. (24)	88.9%	50%	72.9%	83.3%	29.2%	41.7%	3	5	B
Nickel et al. (2)	100%	88.9%	93.8%	94.4%	79.2%	83.3%	6	6	A
Thomas et al. (6)	100%	81.0%	91.0%	100%	78.6%	92.9%	6	6	A
Roditi et al. (33)	94.4%	55.6%	72.9%	94.4%	37.5%	100%	4	6	B

#### 3.2.4 Quality assessment results of expert consensus

The expert consensus (27) fulfilled all six quality assessment criteria and demonstrated excellent overall performance (Refer to Table 6). Specifically, it featured transparent sourcing of opinions, involvement of authors with recognized expertise in the field, a clear focus on the interests of the target population, and conclusions derived through systematic analytical processes supported by relevant literature. Furthermore, it provided logically sound justifications for positions diverging from existing evidence. This indicates that the consensus is not only authoritative and reliable but also developed through a rigorous and transparent process, making it suitable for inclusion as high-quality evidence in this study.

**Table 6:** Quality evaluation results of expert consensus (*n* = 1).

Evaluation items	Thompson et al. (27)
1. Is the source of the opinion clearly identified?	Yes
2. Does the source of the opinion have a standing in the field?	Yes
3. Are the interests of the relevant population the central focus of the opinion?	Yes
4. Is the stated position the result of an analytical process?	Yes
5. Is there reference to the extant literature?	Yes
6. Is any incongruence with the literature/sources logically defended?	Yes

#### 3.2.5 Summary of evidence

As presented in Table 7, a total of 41 key evidence-based recommendations were identified in this study, categorized into four domains: preventive approaches, early detection and surveillance, intervention strategies, and system-level management and enhancement. The quality of evidence spans JBI levels 1 to 5, with most recommendations classified as level A, indicating strong consensus. These findings offer a robust, evidence-informed framework for managing perioperative peripheral intravenous fluid extravasation, encompassing risk evaluation, standardized cannulation procedures, continuous monitoring, prompt emergency protocols, and organizational learning through incident analysis and systemic refinement. Central to these recommendations is the goal of minimizing complications and enhancing patient safety by optimizing venous access decisions, reinforcing real-time intraoperative surveillance, applying medication-specific preventive measures, and fostering a culture of safety reporting and continuous improvement.

**Table 7:** Summary of evidence of peripheral venous infiltration/extravasation in adult surgery.

Category	Evidence content	Level	Strength	Applicability
Preventive strategies				
Catheterization plan	In most intraoperative scenarios (such as emergency, resuscitation, and surgery), securing a stable peripheral venous route is of critical importance. With close surveillance, brief-term delivery of vasoactive medications (e.g., less than 6 h) via a sizable and securely placed peripheral vein offers a beneficial balance between potential risks and therapeutic outcomes for prompt circulatory support (20, 23, 33).	1	A	Extravasation
	For patients planning to receive systemic anti-cancer drug therapy, it is recommended to place a central venous access device. For patients with solid tumors, the use of an implantable venous access port (CV port) may be superior to a peripherally inserted central catheter (PICC) or peripheral venous catheterization (26).	2	B	Extravasation
	To prevent potential drug interactions and excessive local vasoconstriction, it is advised to administer only a single type of vasoconstrictor during each instance of peripheral venous access (19).	1	A	Extravasation
Catheters and sites	Catheterization should primarily utilize upper limb veins, ideally selecting those with a diameter exceeding 4 mm. Lower limb veins may be considered only if upper limb access is unfeasible and there is an urgent clinical requirement; however, it is crucial to recognize that their use is associated with a substantially higher risk of thrombophlebitis (19, 20, 22, 24, 33).	1	A	General
	In adult surgical procedures, the selection of peripheral venous puncture sites can be flexibly adjusted according to specific clinical conditions, but areas with joint flexion, such as the wrist and elbow, should be avoided. Choosing sites closer to the trunk (proximal positions) can reduce the risk of extravasation (2, 22, 23, 26, 27, 33).	1	A	General
	Use a catheter with the minimal possible diameter and shortest required length. Avoid inserting the catheter into limbs affected by burns, joint flexion, infection, or lymphedema, as well as those with blood pressure monitoring cuffs, arteriovenous fistulas, or surgical wounds (2, 19, 21, 24).	5	A	General
Catheterization and techniques	Operators should complete rigorous training and successfully pass evaluations prior to conducting puncture procedures on patients. For individuals anticipated to present challenges during puncture—such as those with obesity or a history of repeated punctures—it is advisable to employ ultrasound- or near-infrared-guided catheterization techniques (2, 21, 27).	5	A	General
	Leakage or extravasation is strongly associated with inadequate dressing fixation. Maintaining a clean, dry, and undamaged dressing is crucial, and minimizing unnecessary or frequent replacements is recommended (8).	1	A	General
	Prior to each infusion, verification of blood return should be performed to confirm catheter patency and proper placement (2, 27, 33).	5	A	General
	Peripheral venous catheters should not be replaced on a routine basis unless clinically indicated (8, 26).	3	A	General
Selection and safe use of infusion devices	For high-risk intravenous medications, including vesicants, vasoactive agents, and insulin, the use of smart infusion pumps equipped with standardized drug libraries is recommended as a primary strategy to minimize medication errors and the occurrence of extravasation (19, 21).	5	A	General
Risk identification	Patients who are older, female, diabetic, have impaired vascular function, suffer from altered consciousness, or are administered foaming agents, vasoactive substances, hypertonic fluids, or therapeutic anticoagulants require careful assessment of associated risk factors. Monitoring frequency should be adjusted upward in accordance with the individual’s risk level (2, 8, 20, 24, 33).	5	A	General
Early identification and monitoring				
Monitoring principles	During the operation, as the patient is unable to express discomfort actively, visual observation and palpation should be relied on for assessment. For such patients, the puncture site should be checked every 1 to 2 h; when infusing foaming agents or vasoactive drugs, the frequency of checks should be further increased, and it is recommended to check after each 2–5 mL push or every 5–10 min (19, 23, 27).	1	A	General
	Key symptoms: Pain (if the patient can express it), redness and swelling, changes in skin temperature, slowed infusion rate, pale skin (2, 8, 20, 24, 33).	5	A	General
Evaluation method	When assessing extravasation, a comparison must be made between both limbs, with the unaffected side serving as a reference to contrast the circumference and color changes of the affected limb. The degree of local skin damage should be objectively and standardly evaluated using the INS extravasation scale and the Common Terminology Criteria for Adverse Events of the National Cancer Institute of the United States (2, 6, 37).	5	A	General
	Electronic infusion pumps are not consistently effective in identifying extravasation, and therefore, their alarm mechanisms should not be solely depended on for early detection (2, 21, 33).	5	A	General
	Monitoring for blood return can aid in the early identification of extravasation and is advised as a standard evaluation practice (26, 33).	4	B	General
Disposal measures				
General processing	Immediate management three-step method: 1. Immediately stop the infusion and disconnect the device. 2. Attempt to aspirate the residual drug. 3. Elevate the affected limb (unless compartment syndrome is suspected) (2, 19, 24, 25, 33).	5	A	General
Local intervention	Cold compress is a fundamental measure for dealing with extravasation/infiltration. Its main function is to constrict blood vessels, alleviate inflammation and pain, and limit the local spread and absorption of the drug. The area of the external application should be larger than the area of the leakage to restrict the range of extravasation/infiltration and prevent tissue necrosis (2, 24, 25, 26, 33).	5	B	General
	For foaming agents and irritant drugs, the application of cold compress and hot compress should be differentiated based on the drug category: Cold compress is recommended: for extravasation caused by alkylating agents (such as cisplatin, cyclophosphamide), anthracyclines (such as doxorubicin), antimetabolites (such as 5-fluorouracil), etc. Hot compress is recommended: for vincristine, etoposide, oxaliplatin, and taxanes (such as paclitaxel, docetaxel, especially when used in combination with hyaluronidase). Hot compress should only be considered when the specific drug is clearly identified (such as vincristine, taxanes in combination with hyaluronidase). It is not recommended to use hot compress alone to handle extravasation. It is conditionally advised to apply the dressing for a duration exceeding 24 h (e.g., 15–20 min per session, 3–4 times daily, over a period of 48–72 h), as opposed to limiting the application to 24 h or less (2, 6, 26).	5	B	Extravasation
Drug-specific treatment (for foaming drug extravasation)	Anthracycline drugs (such as doxorubicin, epirubicin) extravasation: It is strongly recommended to use dexrazoxane as an antidote. The infusion should be started within 6 h and administered over 3 days based on body surface area (1,000 mg/m^2^ on days 1–2 and 500 mg/m^2^ on day 3) (6, 26).	2	A	Extravasation
	Paclitaxel (paclitaxel, docetaxel) extravasation: It is recommended to use hyaluronidase as an antidote, which can be subcutaneously injected around the extravasation area within 3–4 h (2, 6, 26).	5	B	Extravasation
	For extravasation of platinum-based drugs involving more than 20 mL of cisplatin and a concentration greater than 0.5 mg/mL, it is recommended to use sodium thiosulfate for local treatment as soon as possible. The 10% or 25% stock solution should be diluted with sterile water to prepare a 1/6 molar solution (approximately 0.167 molar). Multiple subcutaneous injections of 0.1 mL each should be administered around the extravasation site, totaling 1 mL, to ensure even distribution. The method for preparing 10 mL of a 1/6 molar solution is as follows: For 25% sodium thiosulfate solution: Mix 1.6 mL of the stock solution with 8.4 mL of sterile water. For 10% sodium thiosulfate solution: Mix 4.0 mL of the stock solution with 6.0 mL of sterile water (6, 25, 26).	5	A	Extravasation
	Vincristine and vinorelbine extravasation: It is recommended to use hyaluronidase as an antidote as soon as possible. Administer 150 units subcutaneously, injecting at five points around the extravasation area (2, 6, 25, 26).	5	A	Extravasation
	Mitomycin C, mitoxantrone, anthracyclines (when dexrazoxane is unavailable): Use dimethyl sulfoxide. It is recommended to start local application within 10–25 min after extravasation. Apply 50–99% externally, once every 8 h for a total of 7 days (6).	5	A	Extravasation
Drug-specific treatment (for high-risk irritant drug extravasation)	When vasoactive drugs (such as norepinephrine and dopamine) extravasate, it is recommended to use phentolamine for local subcutaneous infiltration as soon as possible within 12 h to counteract their vasoconstrictive effects. If phentolamine is not available, terbutaline can be considered as an alternative. The specific operation is as follows: dissolve 5 to 10 mg of phentolamine in 5 mL of 0.9% sodium chloride solution and perform local circular block; or apply 2% nitroglycerin ointment to the extravasation site and a 2 to 3 cm range around it, and repeat every 8 h according to the clinical response (19, 20, 23, 25).	2	A	Extravasation
	The suggested dilution ranges for norepinephrine are 16–64 μg/mL, while those for phenylephrine fall between 40 and 400 μg/mL. Implementing standardized dilution procedures may help minimize the likelihood of extravasation (23).	1	A	Extravasation
Drug-specific treatment (for infiltration of other irritant and non-foaming agents)	Irritant drug extravasation (such as some antibiotics, electrolyte solutions, propofol): Generally, no special antidote is required. Cold compress, elevation of the affected limb and monitoring are the main measures. Hyaluronidase can be used when necessary to promote absorption (2, 6, 24, 25, 33).	5	A	Extravasation
Other	In the absence of a specific antidote, saline irrigation may serve as an alternative intervention within the first hour (24, 25, 33).	5	B	General
	It is not recommended to manage extravasation of anticancer drugs by local injection of corticosteroids such as dexamethasone, hydrocortisone, methylprednisolone (26).	5	B	Extravasation
Surgical intervention	Infiltration: Removal of non-necrotic tissue through debridement is not advised (26).	3	B	Infiltration
	When the tissue damage is severe, the condition progresses rapidly or there is a suspicion of compartment syndrome, a surgical consultation should be immediately invited to assess whether timely surgical interventions such as debridement, moist wound healing treatment or flap reconstruction are necessary (2, 24, 25, 33)	5	A	General
	If the extravasate is corrosive or belongs to high-risk corrosive anti-tumor drugs, for such high-risk groups, conservative treatment should not be relied on alone. Instead, timely referral to the surgical department or more active intervention measures should be taken when necessary to upgrade the overall treatment strategy (6). Note: High-risk patients - such as those currently using DNA-binding thrombolytic drugs, patients estimated to have a large amount of extravasation, and those meeting the grade 2, 3 or 4 extravasation criteria of the Common Terminology Criteria for Adverse Events of the National Cancer Institute of the United States - may be more suitable for surgical consultation or upgraded treatment intervention (37).	5	B	Extravasation
System management and improvement				
Evaluation and follow-up framework	Objective evaluation parameters: redness, edema, measurement of extravasation or leakage extent, range of motion in proximal and distal joints, overall joint mobility, capillary refill duration, skin paleness, presence of blisters, ulcer development, additional dermal alterations, and pigmentation changes (2, 6, 27).	5	A	General
	Subjective assessment indicators include: changes in abnormal sensations compared with the baseline, the degree of pain, alterations in joint range of motion, and the impact on daily function and quality of life (2, 6, 27).	5	A	General
	Recorded information includes: medication name, site of extravasation, type of vascular access device, estimated volume of leaked substance, measurements of the affected area, interventions performed, patient’s response to treatment, institution-specific extravasation grading or scoring system, photographs taken in accordance with organizational guidelines, and documentation of patient or caregiver education completion (2, 6, 27, 33).	5	A	General
	Key aspects of patient education: Employ the “teach-back” method as a best practice, combining verbal instruction with written materials to ensure understanding. Instruct patients and caregivers to maintain cleanliness and dryness of the affected site, refrain from touching the area, and be mindful of appropriate clothing choices. Adhere strictly to the prescribed topical treatment regimen and attend follow-up visits as scheduled by the healthcare provider. Do not apply any topical creams or moisturizers without explicit guidance from the medical team. Monitor the site closely for signs of local reaction—including redness, pain, blistering, peeling, or linear (cord-like) changes—and promptly notify healthcare professionals if any of these occur. Additionally, report any delayed complications following extravasation, particularly if fever reaches or exceeds 38.0 °C (100.4 °F) (2, 6, 27).	5	A	General
	Follow-up arrangement suggestions: The follow-up time and intervals after infiltration/extravasation vary greatly in the literature. It is recommended to initiate follow-up within 1 day (on-site or remote), and continue for several weeks to several months based on the treatment response and the nature of the drug. For DNA-binding vesicular drug extravasation, as the symptoms usually appear later, it is recommended to follow up for at least 3 weeks. If referred to a specialist (such as plastic surgery, wound care), ensure there is a clear follow-up plan (2, 6).	5	A	General
Adverse event report	Uniformly document incidents of infiltration/extravasation, including details such as the medication involved, dosage, and actions taken, and submit reports to the hospital’s adverse event reporting system ((19); (27); (2); (33)).	5	A	General
Quality improvement	Continuous improvement of clinical practice should be achieved through strengthening professional training for nursing staff in the prevention and management of infiltration/extravasation, combined with root cause analysis, teamwork among the nursing team, and regular quality reviews, in order to effectively reduce the incidence of such events (2, 24, 25, 27).	5	A	General
Teamwork	Foster interdisciplinary team development, collaborative decision-making, and the establishment of a safety-oriented culture (2, 25).	5	A	General

## 4 Discussion

### 4.1 Key findings and evidence analysis

Based on the existing research evidence, the prevention and control of peripheral venous Infiltration or extravasation during surgery should cover the entire process of prevention, early detection, standardized treatment, and system optimization. In terms of preventive strategies, sufficient clinical evidence supports the optimization of venous access selection. It is recommended to prioritize the use of veins in the proximal part of the upper limb with a diameter greater than 4 mm, avoid areas with joint movement, and use the thinnest possible catheter type to reduce the risk of complications. This conclusion is consistent with the research of Fan et al., who also pointed out that the puncture site is one of the important factors affecting the occurrence of extravasation (22). For patients scheduled to receive high-risk drug infusions (such as chemotherapy drugs or vasoactive drugs), current evidence suggests that the venous access requirements should be evaluated in advance, and central venous access should be established as a priority when necessary to effectively avoid severe extravasation events (23, 26).

Regarding intraoperative monitoring, relevant studies consistently emphasize the importance of active and frequent rounds. Since patients are in a state without the ability to express themselves during surgery, medical staff become the sole key observers for identifying abnormalities (19, 23). Guidelines recommend routine checks every 1 to 2 h; when vasoactive drugs are being administered, the frequency should be shortened to every 5 to 10 min or adjusted dynamically based on the dosage, which is a core measure for intraoperative monitoring. This 5–10 min recommendation is derived from expert consensus (Level 5 evidence) rather than randomised controlled trials. In real-world operating rooms, such frequent checks may be challenging during lengthy or high-acuity procedures; therefore, a risk-stratified approach is more practical – every 5–10 min for high-risk infusions (vasoactive drugs, vesicants) and every 1–2 h for standard infusions, with infusion pump alarms serving as adjuncts, not replacements. Additionally, multiple studies clearly warn that the alarm function of infusion pumps should not be relied upon as the sole indicator for the occurrence of extravasation, as it has limitations in terms of sensitivity and timeliness (2, 21).

At the clinical management level, evidence generally supports the standardized initial response process of “stopping the infusion - withdrawing the residual drug solution - elevating the affected limb” for all extravasation events (2, 19, 25). More importantly, individualized intervention measures based on the pharmacological and tissue damage mechanisms of different types of drugs should be adopted for extravasation caused by different drugs. For example, phentolamine can be applied locally to antagonize extravasation of vasoactive drugs, while dexrazoxane is recommended as a specific antidote for anthracycline chemotherapy drug extravasation. Such targeted treatment strategies have been proven to be of great significance in reducing tissue damage and improving clinical outcomes (19, 20, 23, 26). When severe tissue damage occurs, the surgical consultation mechanism should be initiated promptly and a clear escalation treatment pathway should be followed to ensure patient safety (2, 6). Interpretive commentary: Early aspiration reduces drug-tissue contact volume, limiting damage. Antidotes (phentolamine, dexrazoxane) have specific mechanisms supported by case series; supportive care suffices only for non-cytotoxic leaks. Timing thresholds: dexrazoxane ≤6 h, phentolamine ≤12 h; delayed antidote use reduces efficacy; supportive care escalation beyond 24-48 h may increase necrosis risk.

### 4.2 Strengths and limitations of the evidence

The strengths of this summary are as follows: First, the included evidence is of a high grade, encompassing multiple recent and high-quality clinical guidelines [such as 6, 39] and expert consensuses, ensuring the authority and timeliness of the recommendations (2, 6, 27). Second, the evidence sources cover both international frontiers and local practices (such as the guidelines of the Chinese Nursing Association), enhancing the applicability of clinical application (25). Third, structured evidence extraction and grading (JBI grading and recommendation strength) were adopted, making the clinical guidance value of the recommendations clearer.

This evidence summary included four types of sources: clinical practice guidelines, systematic reviews, evidence summaries/clinical decisions, and expert consensus. While this approach ensured broad coverage of available evidence, the heterogeneity across these source types inevitably influences the strength and interpretation of the recommendations. Guidelines and high-quality systematic reviews underwent rigorous development processes and thus provided the most robust, directly actionable recommendations. In contrast, expert consensus and clinical decision articles with lower methodological scores were used as supplementary support, with their limitations explicitly noted when cited. Therefore, readers should prioritise recommendations derived from guidelines and high-quality systematic reviews, while exercising caution when applying suggestions from lower-level evidence types. The evidence grading (JBI levels 1–5) and recommendation strength (Level A) reported in Table 7 further help distinguish the reliability of individual recommendations. Future updates should aim to include more high-quality primary studies to reduce reliance on heterogeneous secondary evidence.

However, this study also has certain limitations: Firstly, although the search was comprehensive, high-quality original research specifically designed for the intraoperative environment is still relatively limited. Some recommendations are based on research evidence from perioperative or ward environments, and their direct applicability needs to be further verified in clinical practice. Secondly, some of the included literature (such as two UpToDate clinical decisions) have insufficient methodological transparency. Although they are highly clinically practical, their robustness as evidence is relatively low (20, 21). Finally, due to differences in drug availability and medical resource allocation among different countries and regions, some recommendations (such as the application of specific antidotes) need to be adjusted in accordance with local conditions when implemented. Fourth, restricting the search to English and Chinese databases may have introduced language bias, as relevant evidence published in other languages could have been missed.

### 4.3 Local practice and evidence-based considerations on lidocaine/procaine combined with glucocorticoid local block and TCM-based adjunctive approaches

In clinical practice in China, lidocaine or procaine combined with glucocorticoid for local infiltration block is commonly used to relieve pain and swelling caused by infusion extravasation. However, none of the high-quality guidelines recommend this method, and current evidence does not demonstrate clear benefits in preventing tissue injury progression or improving long-term outcomes (2, 28–31, 32). Standard physical measures, such as cold or warm compresses, remain the core recommended interventions (2). Moreover, for cytotoxic drug extravasation, local injection may aggravate tissue damage or promote drug spread; therefore, routine use of this block therapy is not recommended.

In addition, several Traditional Chinese Medicine (TCM)-based external therapies have been reported as potential adjunctive strategies for extravasation-related soft tissue injury in China. While these TCM-based interventions (e.g., herbal external application, integrative wound-care) are described in local case reports, their evidence level remains low (case series or observational reports) (24). As such, their use should also be confined to adjunctive settings under careful assessment and full informed consent.

Therefore, both the local anesthetic–glucocorticoid block and TCM external therapies should be regarded as optional adjunctive measures only, limited to non-vesicant or low-irritant extravasation, and applied only after professional evaluation with detailed documentation. The foundation of extravasation management remains standard care: immediate cessation of infusion, catheter removal, aspiration of residual fluid if possible, limb elevation, and thermal compresses (cold/warm), with escalation (e.g., surgical consult) guided by clinical severity rather than volume of extravasation (32, 33).

### 4.4 Implications for clinical practice

1. Establish standardized procedures: Medical institutions should develop or update their “Intraoperative Peripheral Venous Infusion Safety Management Regulations” based on the core recommendations of this summary, institutionalizing prevention strategies, monitoring frequencies, grading assessment tools (such as the INS extravasation scale), and standardized handling procedures.
2. Strengthen team training and capacity building: Specialized training should be provided for anesthesiologists and operating room nurses, covering the identification of high-risk patients, ultrasound/NIR vascular visualization-guided catheterization techniques, early signs of extravasation/leakage, and indications and methods for the use of specific antidotes. Training should emphasize simulation exercises and case analysis.
3. Optimize technology and human defense: Actively introduce and rationally apply auxiliary technologies such as near-infrared vascular imaging and intelligent infusion pumps, but it must be clear that technology is an auxiliary tool and cannot replace the active and frequent bedside assessments by medical staff.
4. Build a safety culture and reporting system: Encourage a non-punitive culture of reporting adverse events, conduct root cause analysis (RCA) for each extravasation/leakage incident, and feed the analysis results back into process optimization and training, forming a closed-loop management of “reporting - analysis - improvement” (2, 25, 27).

### 4.5 Integrated practice framework: standardized management process recommendations

**Figure 2:**
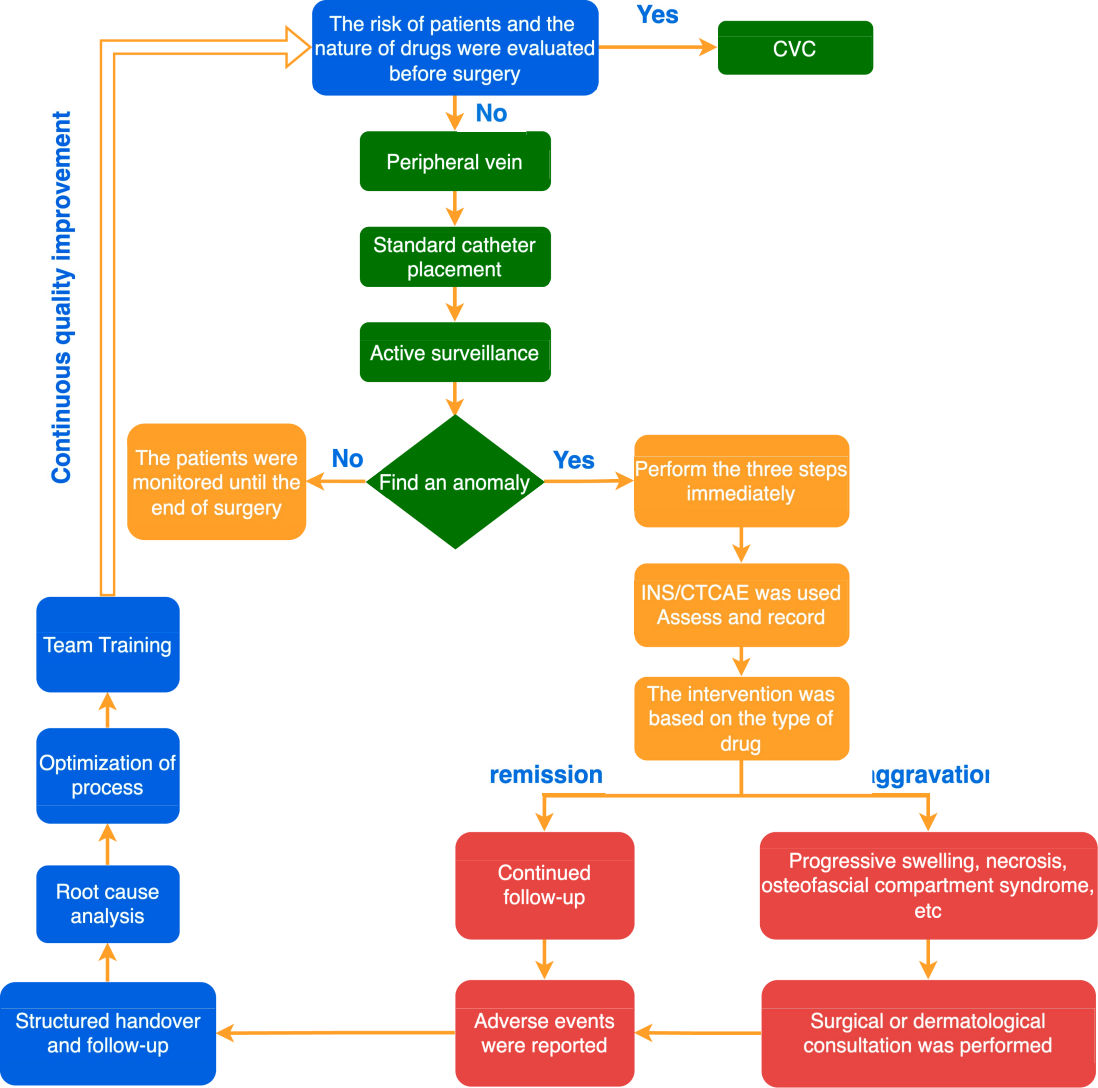
Standardized nursing procedure flowchart for intraoperative fluid infiltration/extravasation.

Based on the above evidence summary and discussion, to transform the best evidence into actionable clinical behaviors, we have developed a “Standardized Process for the Prevention and Management of Peripheral Intravenous Fluid Extravasation/Leakage during Surgery” (Figure 2). This process integrates a full-cycle closed-loop management from preoperative assessment to systematic learning, aiming to provide a clear decision-making path and action guidance for the operating room team. The process particularly emphasizes the core position of active monitoring and specific interventions based on drug categories. We suggest that medical institutions can adapt this process framework to their local conditions and form specific operational norms and checklists.

### 4.6 Suggestions for future research

Future research could focus on the following directions: ① Conduct prospective cohort studies on intraoperative patients (especially those undergoing long surgeries or receiving high-risk drug infusions) to more accurately determine the incidence of intraoperative complications and independent risk factors. ② Design and validate new monitoring technologies or tools suitable for the intraoperative environment, which are efficient and feasible (such as wearable sensors, continuous tissue pressure monitoring). ③ Carry out high-quality comparative effectiveness studies to evaluate the cost-effectiveness of different preventive interventions (such as different dressing fixation techniques, different catheter materials) in intraoperative scenarios. ④ Explore artificial intelligence-based predictive models for preoperatively identifying patients at extremely high risk of peripheral venous leakage or extravasation.

## 5 Conclusion

This evidence summary indicates that the effective prevention and control of peripheral intravenous fluid extravasation during surgery should be based on an integrated strategy that combines “prevention - identification - treatment - systematic learning.” The key to prevention lies in careful venous assessment and selection, standardized catheterization techniques, and the pre-identification of high-risk drugs and patients. The core of intraoperative monitoring is the regular and proactive inspection by medical staff, with any technical equipment serving only as an auxiliary. The first step in treatment is to immediately implement the “stop - aspirate - elevate” three-step method, and then quickly initiate differentiated physical interventions or specific detoxification treatments based on the characteristics of the extravasated drug. Integrating this evidence into systematic clinical practice is essential to enhance intraoperative infusion safety. Looking forward, we strongly advocate for widespread adoption of this framework and for future research to validate its components, ultimately establishing a new benchmark for preventable harm reduction in perioperative care.
